# HMGB1 Regulates Adipocyte Lipolysis via Caveolin-1 Signaling: Implications for Metabolic and Cardiovascular Diseases

**DOI:** 10.3390/ijms26094222

**Published:** 2025-04-29

**Authors:** Julia Chu-Ning Hsu, Kuan-Ting Chiu, Chia-Hui Chen, Chih-Hsien Wang, Song-Kun Shyue, Tzong-Shyuan Lee

**Affiliations:** 1 Department of Veterinary Medicine, College of Veterinary Medicine, National Chung Hsing University, 145, Xingda Road, South District, Taichung 402202, Taiwan; juliacnhsu@dragon.nchu.edu.tw; 2 Department of Physiology, School of Medicine, National Yang-Ming University, 155, Sec. 2, Linong Street, Beitou District, Taipei 112304, Taiwan; 3 Graduate Institute and Department of Physiology, College of Medicine, National Taiwan University, 1, Sec. 1, Jenai Road, Zhongzheng District, Taipei 100233, Taiwan; 4 Cardiovascular Surgery, Department of Surgery, National Taiwan University Hospital and College of Medicine, 7, Chungshan South Road, Zhongzheng District, Taipei 100225, Taiwan; 5 Institute of Biomedical Sciences, Academia Sinica, 128, Sec. 2, Academia Road, Nankang District, Taipei 115201, Taiwan

**Keywords:** high-mobility group box 1, adipogenesis, lipolysis, caveolin-1, obesity-related metabolic disorders

## Abstract

High-mobility group box 1 (HMGB1) is a nuclear protein that can be secreted or released into the extracellular environment during cellular stress, functioning as a damage-associated molecular pattern molecule. This study investigates the role of HMGB1 in adipocyte development and metabolism, explicitly examining its interaction with β3-adrenergic receptor-mediated lipolysis and caveolin-1 (CAV1) regulation, which may influence cardiovascular risk factors. Using 3T3-L1 preadipocytes and mouse embryonic fibroblasts, we demonstrated that HMGB1 expression increases progressively during adipogenesis, reaching peak levels in mature adipocytes. While exogenous HMGB1 treatment did not affect preadipocyte proliferation or differentiation, it inhibited lipolysis in mature adipocytes. Mechanistically, HMGB1 suppressed β3-adrenergic receptor agonist CL-316,243-induced hormone-sensitive lipase activation by reducing protein kinase A-mediated phosphorylation and attenuating extracellular signal-regulated kinase signaling without affecting upstream cyclic AMP levels. We discovered a novel regulatory mechanism wherein CAV1 physically interacts with HMGB1 in mature adipocytes, with c-Src-dependent CAV1 phosphorylation functioning as a negative regulator of HMGB1 secretion. This finding was confirmed in CAV1-deficient models, which displayed increased HMGB1 secretion and diminished lipolytic activity both in vitro and in vivo. Furthermore, administering HMGB1-neutralizing antibodies to wild-type mice enhanced fasting-induced lipolysis, establishing circulating HMGB1 as a crucial antilipolytic factor. These findings reveal HMGB1’s previously uncharacterized role in adipose tissue metabolism as a negative regulator of lipolysis through CAV1-dependent mechanisms. This work provides new insights into adipose tissue metabolism regulation and identifies potential therapeutic targets for obesity-related metabolic disorders and cardiovascular diseases.

## 1. Introduction

High-mobility group box 1 (HMGB1) is a highly conserved nuclear protein that regulates gene expression by binding to DNA [1,2,3,4,5]. As an architectural chromatin-binding protein, it bends DNA strands, enhancing accessibility to transcription factors and other regulatory proteins [4,5,6]. This structural role facilitates interactions between DNA and essential regulatory elements, promoting gene activation [5]. However, during cellular stress or injury, HMGB1 can be actively secreted by stressed immune cells or passively released from dying cells into the extracellular space [3,4]. There, it functions as a damage-associated molecular pattern molecule, triggering and sustaining inflammatory responses [1,4]. Upon release into the extracellular space, HMGB1 drives inflammation by engaging pattern recognition receptors, particularly toll-like receptor 4 (TLR4) and the receptor for advanced glycation end products (RAGE) [1,2,3,4,5]. This activation triggers intracellular signaling cascades that regulate transcription factors such as nuclear factor-κB, sterol regulatory element-binding protein 1 (SREBP-1), peroxisome proliferator-activated receptor γ (PPARγ), and CCAAT/enhancer-binding protein β, while also modulating AMP-activated protein kinase (AMPK) activity [7,8,9]. The resulting signaling promotes the production and release of proinflammatory mediators, including cytokines, chemokines, and adhesion molecules [2,7,9]. Persistent HMGB1 release and prolonged receptor activation can contribute to chronic inflammation, marked by sustained immune cell recruitment and continuous production of inflammatory mediators [1,2,4,5]. In obesity, elevated extracellular HMGB1 establishes a self-perpetuating inflammatory cycle in adipose tissue, where ongoing receptor signaling maintains inflammatory pathway activation in both adipocytes and resident immune cells [3,10,11]. This chronic state disrupts adipose tissue function, further exacerbating obesity-associated inflammation [2,10,11].

Obesity is marked by chronic low-grade inflammation and persistent immune activation in adipose tissue, which disrupts metabolic regulation and contributes to adipocyte insulin resistance and type 2 diabetes [3,12]. As adipose tissue expands through both adipocyte hypertrophy and hyperplasia, cellular stress, adipose tissue hypoxia, and adipocyte death trigger both the passive release of HMGB1 from necrotic adipocytes and its active secretion from stressed adipocytes [3,10,11,12,13]. This extracellular HMGB1 initiates a self-perpetuating inflammatory cycle in adipose tissue by binding to RAGE and TLR4 receptors, promoting macrophage recruitment and polarization from an anti-inflammatory M2 to a proinflammatory M1 phenotype within adipose tissue [1,3,4,5,12]. Activated adipose tissue macrophages secrete inflammatory cytokines such as tumor necrosis factor α and interleukin-1β, as well as additional HMGB1, which further sustains chronic adipose inflammation [3,12]. HMGB1-driven inflammation impairs insulin signaling in adipocytes and is associated with an increased risk of obesity-related complications, including cardiovascular disease and certain cancers [2,4,5,11]. By promoting atherosclerosis, arterial stiffness, and hypertension, obesity-induced chronic inflammation contributes to cardiovascular disease [14,15]. The role of HMGB1 in perpetuating inflammation exacerbates these conditions, making it a potential biomarker for cardiovascular risk. Extracellular HMGB1 has been shown to enhance caveolin-1-dependent receptor-mediated endocytosis significantly [16].

Caveolin-1 (CAV1), a crucial structural protein, plays essential roles in cellular signal transduction and cholesterol transport [17]. During the differentiation of monocytes into macrophages, CAV1 expression is upregulated, potentially contributing to heightened immune responses [18]. The interaction between HMGB1 and CAV1 may represent a key mechanistic pathway linking systemic inflammation to adipose tissue dysfunction. Specifically, CAV1-dependent changes in adipocyte membrane dynamics promote the release of pro-inflammatory cytokines, thereby perpetuating the chronic inflammatory environment characteristic of various cardiometabolic disorders [19,20].

Despite growing evidence linking HMGB1 to metabolic regulation, the intricate interplay among HMGB1, adipose tissue expansion, and CAV1 remains incompletely elucidated. Understanding these interactions is crucial for unraveling the molecular mechanisms underlying adipose tissue homeostasis and dysfunction. Therefore, this study was conducted, first, to characterize the mechanistic involvement of HMGB1 in adipocyte differentiation, maturation, and lipid metabolism; second, to investigate the functional relationship between HMGB1 and β3-adrenergic receptor-mediated lipolytic signaling pathways, with a particular focus on key downstream effectors; and third, to explore the dynamic molecular crosstalk between HMGB1 and CAV1 throughout adipogenic development, particularly during states of heightened lipolytic activity. These insights may reveal novel regulatory nodes that contribute to adipose tissue remodeling and metabolic adaptation in response to physiological and pathological stimuli.

## 2. Results

### 2.1. HMGB1 Expression Is Increased During 3T3-L1 Adipocyte Differentiation

**Figure 1:**
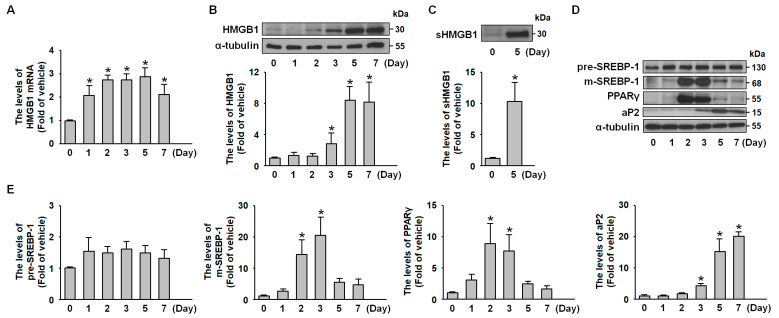
**HMGB1 expression increased in parallel with the expression of adipogenic markers during 3T3-L1 adipogenic differentiation.** During adipogenic differentiation, 3T3-L1 preadipocytes were harvested for mRNA, protein, and culture medium collection at the indicated time points. (**A**) HMGB1 mRNA levels were assessed using RT-PCR. (**B**) Intracellular HMGB1 and α-tubulin protein levels were analyzed using western blotting. (**C**) Secreted HMGB1 (sHMGB1) protein levels in the culture medium were measured using western blot analysis. (**D**) Western blot analysis of pre-SREBP-1, m-SREBP-1, PPARγ, aP-2, and α-tubulin protein levels. (**E**) Quantitative results of panel D. All data are presented as mean ± SEM from four independent experiments. * *p* < 0.05 versus the 0 min time point.

To investigate HMGB1 expression dynamics during adipogenesis, we monitored the complete differentiation process from preadipocytes to mature adipocytes. Western blot and reverse transcriptase-PCR (RT-PCR) analyses of whole-cell lysates revealed a progressive increase in HMGB1 mRNA (Figure 1A) and protein levels (Figure 1B), with preferential expression in mature adipocytes. Additionally, secreted HMGB1 (sHMGB1) was detected in the culture medium when mature adipocytes were maintained under serum-free conditions for 24 h (Figure 1C). Using the established 3T3-L1 preadipocyte model, we explored the relationship between HMGB1 and adipogenesis, with successful differentiation confirmed by the time-dependent induction of key transcriptional factors and adipocyte markers, including SREBP-1, PPARγ, and adipocyte fatty acid binding protein (aP-2) (Figure 1D,E).

### 2.2. Exogenous HMGB1 Does Not Affect Adipogenesis, Including Preadipocyte Proliferation and Differentiation

**Figure 2:**
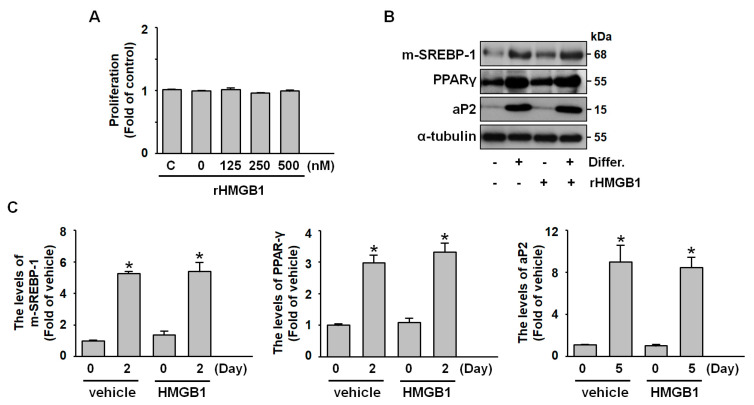
**Recombinant HMGB1 treatment does not affect preadipocyte proliferation or adipogenic differentiation.** (**A**) 3T3-L1 preadipocytes were treated with various concentrations of recombinant HMGB1 (rHMGB1; 0, 125, 250, and 500 nM) for 24 h, and cell proliferation was assessed using the MTT assay. (**B**) Western blot analysis of m-SREBP-1, PPARγ, aP-2, and α-tubulin during adipogenic differentiation. (**C**) Quantitative results of panel B. All data are presented as mean ± SEM from four independent experiments. * *p* < 0.05 versus the vehicle group.

To investigate the role of HMGB1 in adipogenesis, we examined both the proliferation and differentiation phases of preadipocyte development. MTT assay results indicated that exogenous HMGB1 administration did not significantly affect preadipocyte proliferation (Figure 2A). Similarly, western blot analysis revealed that HMGB1 treatment did not alter the expression patterns of key differentiation markers, including the mature form of SREBP-1, PPARγ, and aP-2 (Figure 2B,C). These findings suggest that HMGB1 does not play a significant role in the adipogenic process within our 3T3-L1 preadipocyte model.

### 2.3. CL-316,243 Induces Adipocyte Lipolysis via PKA- and ERK-Mediated HSL Activation

**Figure 3:**
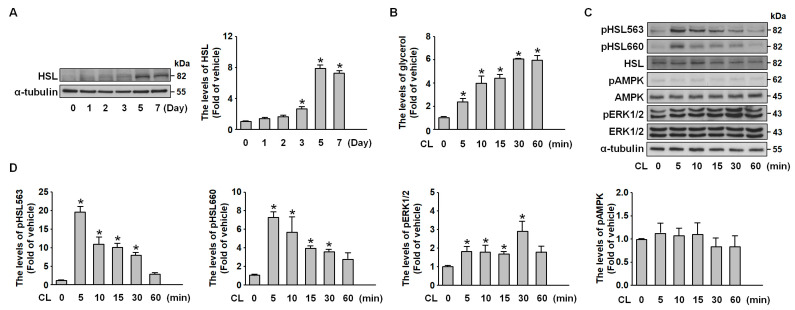
**CL-316,243 induces lipolysis in differentiated 3T3-L1 adipocytes via β3-adrenoreceptor activation.** (**A**) Western blot analysis of HSL and α-tubulin during 3T3-L1 adipogenic differentiation. (**B**,**C**) Differentiated 3T3-L1 adipocytes were treated with CL-316,243 (1 µM), a β3-adrenoreceptor agonist, for the indicated time points (0, 5, 10, 15, 30, and 60 min). (**B**) Glycerol release into the culture medium was quantified using a colorimetric assay. (**C**) Western blot analysis of HSL phosphorylation at Ser563 and Ser660, total HSL, phosphorylated AMPK, total AMPK, phosphorylated ERK1/2, total ERK1/2, and α-tubulin. (**D**) Quantitative results of panel C. All data are presented as mean ± SEM from four independent experiments. * *p* < 0.05 versus the 0 min time point.

We confirmed that hormone-sensitive lipase (HSL), the rate-limiting enzyme in lipolysis, is expressed in mature adipocytes (Figure 3A). HSL catalyzes key hydrolysis steps from triglycerides to monoglycerides, with its activity primarily regulated by multiple phosphorylation sites [21,22]. To investigate these regulatory mechanisms, we stimulated adipocytes with CL-316,243 (1 μg/mL), a selective β3-adrenoreceptor agonist known to activate extracellular signal-regulated kinase (ERK) and protein kinase A (PKA) signaling pathways [23]. Glycerol release analysis revealed that CL-316,243 induced a transient lipolytic response (Figure 3B). HSL phosphorylation at Ser563 and Ser660 occurred rapidly, peaking at 5 min post-stimulation, indicating PKA-mediated activation (Figure 3C,D). Additionally, ERK phosphorylation was observed at 30 min, while AMPK phosphorylation remained unchanged (Figure 3C,D). These findings suggest that CL-316,243 promotes adipocyte lipolysis by activating HSL through distinct phosphorylation events at Ser563 and Ser660, alongside ERK pathway activation.

### 2.4. HMGB1 Suppresses Adipocyte Lipolysis by Inhibiting PKA-HSL Phosphorylation and ERK Activation

**Figure 4:**
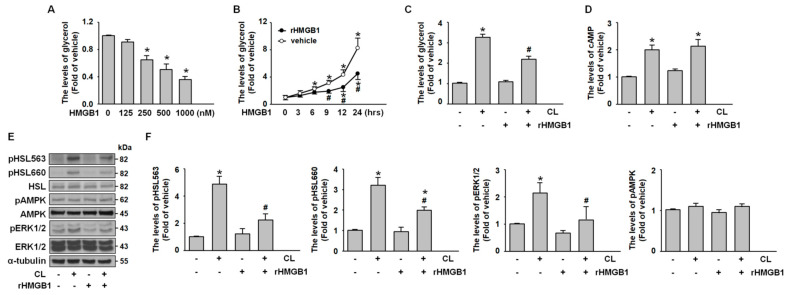
**HMGB1 inhibits CL-316,243-induced lipolysis by reducing HSL and ERK phosphorylation.** (**A**,**B**) After 7-day of differentiation, 3T3-L1 adipocytes were treated with (**A**) various concentrations of recombinant HMGB1 (rHMGB1; 0, 125, 250, 500, and 1000 nM) or (**B**) 500 nM HMGB1 for the indicated times (0, 3, 6, 9, 12, and 24 h). Glycerol release into the culture medium was measured. (**C**,**D**) 3T3-L1 adipocytes were pretreated with 500 nM HMGB1 for 30 min, followed by stimulation with CL-316,243 for 1 h (**C**) or 5 min (**D**). (**C**) Glycerol release into the culture medium was assessed. (**D**) Intracellular cAMP levels were measured using ELISA. (**E**) 3T3-L1 adipocytes were pretreated with 500 nM HMGB1 for 30 min, followed by CL-316,243 treatment for 5 or 30 min. Western blot analysis was performed to assess HSL phosphorylation at Ser563 and Ser660, total HSL, phosphorylated AMPK, total AMPK, phosphorylated ERK1/2, total ERK1/2, and α-tubulin. (**F**) Quantitative results of panel E. All data are presented as mean ± SEM from four independent experiments. * *p* < 0.05 versus the 0 min time point or vehicle group. # *p* < 0.05 versus the CL-316,243-treated group.

To study the effect of HMGB1 on adipocyte metabolism, differentiated adipocytes were exposed to recombinant HMGB1 (rHMGB1) under serum-free, low-glucose conditions. Glycerol release measurements indicated that rHMGB1 suppressed starvation-induced lipolysis in a time- and dose-dependent manner (Figure 4B). To investigate the underlying mechanism, adipocytes were pretreated with rHMGB1 (500 nM, 30 min) before stimulation with CL-316,243. Analysis of the culture medium revealed that rHMGB1 markedly inhibited CL-316,243-induced glycerol release (Figure 4C). While enzyme-linked immunosorbent assay (ELISA) showed no effect of rHMGB1 on CL-316,243-induced cyclic AMP (cAMP) elevation (Figure 4D), western blot analysis demonstrated that rHMGB1 attenuated both PKA-mediated HSL phosphorylation and ERK activation (Figure 4E,F). Notably, AMPK signaling remained unaffected. These findings suggest that HMGB1 exerts antilipolytic effects in 3T3-L1 adipocytes by inhibiting PKA and ERK signaling cascades without altering upstream cAMP levels or AMPK activity.

### 2.5. Src Signaling Mediates CAV1-HMGB1 Binding and Inhibits HMGB1 Release

**Figure 5:**
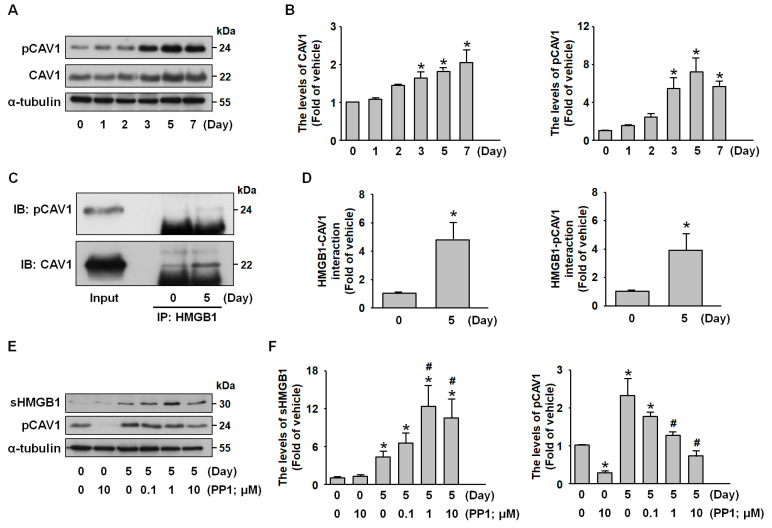
**Role of c-Src/CAV1 signaling in HMGB1 secretion from 3T3-L1 adipocytes.** (**A**) Western blot analysis of CAV1, phosphorylated CAV1 (pCAV1), and α-tubulin during 3T3-L1 preadipocyte differentiation. (**B**) Quantitative results of panel (**A**). (**C**) Immunoprecipitation of cell lysates from undifferentiated and 5-day differentiated 3T3-L1 adipocytes with an anti-HMGB1 antibody, followed by immunoblotting with anti-pCAV1, CAV1, and HMGB1 antibodies. (**D**) Quantitative results of panel (**C**). (**E**) Undifferentiated and 5-day differentiated 3T3-L1 adipocytes were treated with increasing concentrations of PP1 (0, 0.1, 1, and 10 µM), a Src kinase inhibitor, for 24 h. Western blot analysis was performed to assess pCAV1 and α-tubulin, as well as secreted HMGB1 (sHMGB1) in the culture medium. (**F**) Quantitative results of panel (**E**). All data are presented as mean ± SEM from four independent experiments. * *p* < 0.05 versus the 0 min time point or vehicle-treated group. # *p* < 0.05 versus the untreated group (without PP1).

Based on the previous findings that CAV1 regulates protein secretion, internalization, and vesicle trafficking [24], our study revealed that during adipocyte differentiation, the protein levels of both CAV1 and its phosphorylated form (pCAV1) were increased in parallel with the protein expression of HMGB1 (Figure 5A,B). Co-immunoprecipitation analysis revealed a stronger interaction between HMGB1 and CAV1 and pCAV1 in mature adipocytes than in preadipocytes (Figure 5C,D). To examine the regulatory role of CAV1 phosphorylation in HMGB1 dynamics, mature adipocytes were treated with PP1, a Src kinase inhibitor, as Src mediates CAV1 phosphorylation [25]. Western blot analysis confirmed that PP1 treatment (1 to 10 µM) effectively reduced pCAV1 levels while concurrently increasing sHMGB1 levels (Figure 5E,F). These findings identify Src-dependent CAV1 phosphorylation as a negative regulator of HMGB1 secretion in adipocytes, revealing a novel mechanism governing HMGB1 trafficking in adipose tissue.

### 2.6. sHMGB1 Suppresses Fat Breakdown In Vitro and In Vivo

**Figure 6:**
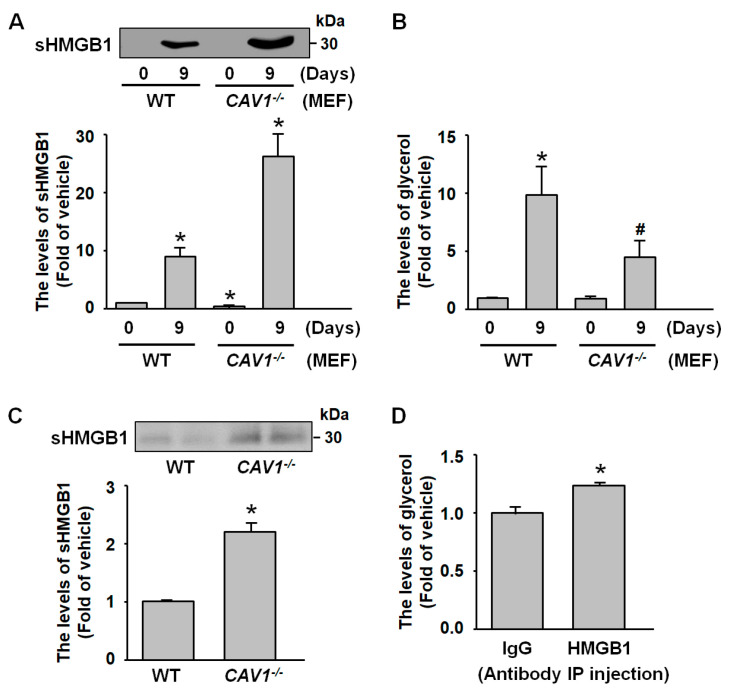
**CAV1 deletion increases HMGB1 secretion and reduces lipolysis.** Mouse embryonic fibroblasts (MEFs) from wild-type (WT) and CAV1-deficient (*CAV1*^−/−^) mice were differentiated into adipocytes for 9 days. (**A**) Western blot analysis of secreted HMGB1 (sHMGB1). (**B**) Glycerol levels were measured as an indicator of lipolysis. (**C**) WT and *CAV1*^−/−^ mice were fasted for 18 h, and serum samples were collected to assess sHMGB1 by western blot analysis. (**D**) WT mice received intraperitoneal injections of either HMGB1 antibody (10 µg) or immunoglobulin G (IgG, 10 µg). After 18-hour fasting, blood samples were collected, and serum glycerol levels were measured. All data are presented as mean ± SEM from four independent experiments. * *p* < 0.05 versus the WT 0-day or IgG group. # *p* < 0.05 versus the WT 9-day group.

Mouse embryonic fibroblasts (MEFs) isolated from caveolin-1-deficient (*CAV1*^−/−^) mice and differentiated for 9 days exhibited increased HMGB1 secretion compared to the MEFs from wild-type (WT) mice, as confirmed by western blot analysis (Figure 6A). Glycerol measurements revealed reduced lipolytic activity in *CAV1*^−/−^ MEFs (Figure 6B), suggesting that CAV1 regulates the secretion from HMGB1 in adipocytes and suppresses fat breakdown. This relationship was further supported in vivo, where fasting serum samples from *CAV1*^−/−^ mice showed higher HMGB1 levels than those in WT mice (Figure 6C). To investigate the physiological role of sHMGB1, WT mice were treated with HMGB1-neutralizing antibodies. After overnight fasting, these mice showed increased lipolysis compared to immunoglobulin G-treated controls (Figure 6D), indicating that circulating HMGB1 acts as an endogenous antilipolytic factor in adipose tissue metabolism.

## 3. Discussion

**Figure 7:**
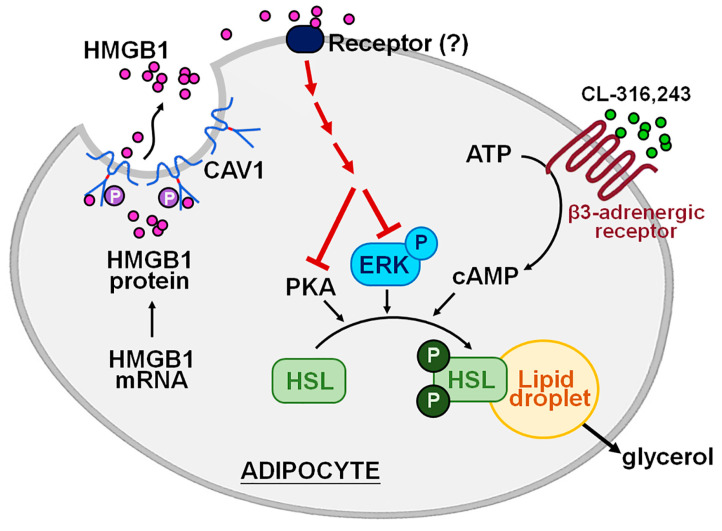
**Schematic illustration of the proposed mechanisms by which HMGB1 regulates lipolysis in adipocytes.** HMGB1 is a key lipid metabolism regulator through the c-Src/CAV1 signaling pathway. HMGB1 does not affect adipogenesis, including preadipocyte proliferation or differentiation, but significantly inhibits lipolysis in mature adipocytes. This inhibition occurs through the suppression of HSL phosphorylation and ERK activation, without altering upstream cAMP signaling, and acts downstream of β3-adrenoreceptor activation. In mature adipocytes, HMGB1 physically interacts with CAV1, whose phosphorylation by Src kinase negatively regulates HMGB1 secretion. As a result, HMGB1 serves as a negative regulator of lipolysis through CAV1-dependent mechanisms, highlighting a potential therapeutic target for obesity-related cardiovascular diseases.

The nuclear protein HMGB1 is known to be actively secreted or passively released extracellularly during cellular stress, serving as a damage-associated molecular pattern [1,4]. In this study, we investigate the physiological role of HMGB1 in adipocyte differentiation and metabolic processes, focusing on its interaction with β3-adrenergic receptor-mediated lipolysis and its interplay with CAV1, which may influence cardiovascular risk factors. We used 3T3-L1 preadipocytes and primary MEFs from WT mice and *CAV1*^−/−^ mice to demonstrate that HMGB1 levels were increased progressively during adipogenesis, peaking in fully differentiated adipocytes. While exogenous HMGB1 did not affect preadipocyte proliferation or differentiation, it inhibited lipolysis in mature adipocytes. HMGB1 suppressed HSL activation at the molecular level by reducing PKA-mediated phosphorylation and ERK pathway signaling without altering upstream cAMP levels. Furthermore, we identified a novel regulatory mechanism in which CAV1 directly interacts with HMGB1 in mature adipocytes, with Src-dependent CAV1 phosphorylation acting as a negative modulator of HMGB1 secretion. This interaction was confirmed in CAV1-deficient models, which showed increased HMGB1 secretion and diminished lipolytic activity both in vitro and in vivo. In addition, applying HMGB1-neutralizing antibodies in WT mice elevated fasting-induced lipolysis, further supporting the role of circulating HMGB1 as an endogenous antilipolytic factor (Figure 7). Our findings reveal HMGB1 as a novel negative regulator of lipolysis in adipose tissue, operating through a CAV1-dependent mechanism, and provide new insights into adipose tissue regulation. These results open potential therapeutic avenues for addressing obesity-related metabolic disorders and cardiovascular diseases.

The critical role of HMGB1 in adipose tissue development has been well-established using HMGB1-deficient mouse models, where pups displayed a complete absence of adipose tissue and died shortly after birth [26]. Our findings, which show progressive increases in HMGB1 expression during adipogenesis, were built on previous research investigating HMGB1’s relationship with adipose tissue function and condition. In lipodystrophic mouse models with compromised subcutaneous fat storage, HMGB1 protein levels are significantly elevated in visceral fat compared to controls [27]. Supporting work by Shimizu et al. demonstrates higher HMGB1 expression in mature adipocytes than in preadipocytes [3], which aligns with our observation of preferential expression in terminally differentiated cells. Following 16 weeks of a high-fat diet, mice show increased HMGB1 expression in epididymal adipose tissue and peripheral blood [28]. In parallel, human studies reveal elevated HMGB1 protein levels in the adipose tissue of obese individuals compared to those with normal weight [10]. Our research identified a correlation between HMGB1 expression and the activation of key adipogenic transcription factors, including SREBP-1, PPARγ, and aP-2, supporting the notion that HMGB1 undergoes various post-transcriptional modifications—acetylation, phosphorylation, glycosylation, and oxidation—despite lacking a conventional secretory signal peptide [4]. Interestingly, our experiments with exogenous HMGB1 administration revealed no significant effects on preadipocyte proliferation or differentiation marker expression, which differs from the findings of Liu et al., who reported substantial HMGB1 effects on cell proliferation and myeloid differentiation in bone marrow mononuclear cells from relapsed acute myeloid leukemia patients [6].

Our findings on HSL expression and its regulation through phosphorylation during CL-316,243-induced lipolysis align with published studies, particularly the rapid phosphorylation of HSL at Ser563 and Ser660 following β3-adrenergic stimulation [29]. Similarly, the ERK phosphorylation observed after CL-316,243 treatment is consistent with results from adrenaline infusion studies, where ERK1/2 phosphorylation increased in parallel with HSL activity [30]. In particular, our observation of unchanged AMPK phosphorylation levels supports observations by Watt et al., who found that epinephrine does not elevate phosphorylated AMPK [31]. The transient lipolytic response with enhanced glycerol release we observed following CL-316,243 administration is also consistent with in vivo findings, where intraperitoneal injection of CL-316,243 (1.0 mg/kg) elevated plasma glycerol levels [32]. Mechanistic investigations revealed that rHMGB1 exerts antilipolytic effects on adipocytes, partially inhibiting β-adrenergic-stimulated lipolysis by targeting specific downstream signaling pathways, such as PKA-mediated HSL phosphorylation and ERK activation, without altering initial cAMP production. These results establish HMGB1 as a key antilipolytic factor, expanding the current understanding of adipocyte regulatory networks and offering new insights into obesity-related cardiovascular diseases associated with dysregulated lipolysis [11,14]. A similar mechanism operates in diabetes mellitus, where HMGB1 contributes to disease progression by activating the TLR4/endothelial nitric oxide synthase signaling pathway [33]. From a different perspective, it has been reported that HMGB1 may attenuate non-alcoholic fatty liver disease through activation of the liver x receptor–α-PPARγ pathway, promoting β-oxidation and relieving endoplasmic reticulum stress [34,35].

Extracellular HMGB1 is well-established as a pro-inflammatory mediator that activates immune responses by engaging pattern recognition receptors, primarily TLR4 and RAGE [1,2,3,4,5]. Within TLR4-mediated signaling, the ATP-binding cassette transporter A1, which plays a critical role in cholesterol efflux, modulates anti-inflammatory responses through PKA activation, thereby contributing to lipid homeostasis in macrophages [36]. Studies have demonstrated that lipopolysaccharide stimulation induces phosphorylation at specific tyrosine residues within the TLR4 receptor complex, with phosphorylation at tyrosine 672 specifically promoting pro-inflammatory signaling via ERK1/2 activation and subsequent c-FOS phosphorylation [37]. Additionally, HMGB1 influences metabolic regulation through RAGE-dependent pathways, as RAGE has been shown to suppress PKA activity while enhancing ERK1/2 signaling in contexts such as airway smooth muscle cell proliferation [38,39]. These findings suggest that TLR4 and RAGE may function as key intermediaries that transduce HMGB1 signals to modulate both PKA and ERK signaling pathways; however, the involvement of this proposed mechanism in HMGB1-mediated regulation in lipolysis requires further investigation.

The interaction between CAV1 and HMGB1 in adipocytes offers valuable insights into the mechanisms governing HMGB1 trafficking, advancing our understanding of its regulatory pathways. Previous studies have shown that CAV1 is actively secreted from adipocytes but not preadipocytes, positioning it as a potential enhancer of adipogenesis [13]. In line with this, our study observed a similar pattern following the administration of HMGB1 to mature adipocytes. Furthermore, our detection of increased HMGB1–CAV1 interaction in mature adipocytes aligns with findings by Scherer et al., who reported that various secretory proteins preferentially associate with CAV1 in terminally differentiated cells [40]. In age-related macular degeneration, the upregulation of both HMGB1 and CAV1 has been implicated in retinal pigment epithelium cell senescence [41]. Additionally, HMGB1 has been shown to facilitate albumin transcytosis through RAGE-dependent Src phosphorylation and CAV1 phosphorylation [42]. In our study, Src inhibition increased sHMGB1 levels and reduced pCAV1 in adipocytes, thereby confirming the role of Src in regulating CAV1 [25]. Identifying Src-dependent CAV1 phosphorylation as a negative regulator of HMGB1 secretion unveils a novel mechanism that can offer new insights into adipose tissue dysfunction in inflammatory conditions, where HMGB1 levels are often dysregulated.

In CAV1-deficient MEFs, HMGB1 secretion was increased, accompanied by reduced glycerol levels, suggesting that the diminished lipolytic activity associated with CAV1 deficiency may be mediated by elevated HMGB1 secretion. The findings from the in vitro study were further investigated in vivo using animal models. When CAV1-deficient mice demonstrate decreased lipolysis, our in vivo findings showed elevated fasting serum HMGB1 levels, implying that increased HMGB1 may contribute to decreased lipolytic activity. The enhanced lipolytic response observed following HMGB1 neutralization provides evidence supporting the role of HMGB1 as an endogenous danger signal that regulates lipid metabolism. Our data reveal a specific divergence in HMGB1 function in adipocytes. Hepatocyte studies suggest that HMGB1 serves as a potent suppressor of hepatic lipid synthesis, protecting against nonalcoholic fatty liver disease through two distinct pathways: sustaining fatty acid β-oxidation processes and alleviating endoplasmic reticulum stress responses [34,35]—effects that stand in opposition to our current observations in adipose tissue. Our results are in line with the findings by Razani et al., who found that CAV1-deficient mice exhibit progressive fat loss, characterized by the depletion of the hypodermal fat layer and a widespread reduction in white adipose tissue [43]. In the human case, the CAV1-deficient patient presents with generalized subcutaneous lipoatrophy, marked by pronounced muscular definition and prominent superficial venous patterns, particularly in the lower extremities [44]. Moreover, clinical examination confirms extensive adipose tissue depletion across subcutaneous, visceral, orbital, and buccal fat depots [45]. As noted, we reported that HMGB1 suppresses lipolysis in adipocytes while Lin et al. found that HMGB1 promotes β-oxidation in hepatocytes [34], suggesting the context-dependent roles of HMGB1 across tissues. CAV1 is known to play essential, yet distinct, roles in liver and adipose tissue [13,34,35,46]. Several lines of evidence have implied the influence of CAV1 expression on the tissue-specific effects of HMGB1 [41,42]. For instance, genetic ablation of CAV1 in the liver leads to dysfunction [46]. In contrast, adipocyte-specific deletion of CAV1 results in defective insulin secretion, diminished insulin-activated protein kinase B signaling, and a partial lipodystrophic phenotype [19]. These findings suggest that CAV1 is a key modulator of metabolic function in a tissue-dependent manner. Although our data indicate a potential interaction between HMGB1 and CAV1 during adipocyte differentiation, whether the biological effects of HMGB1 are directly dependent on CAV1 expression levels remains unclear. Other tissue-specific factors, such as differences in receptor expression or signaling environments, may also contribute to the functional divergence of HMGB1. Collectively, these findings further support the notion that HMGB1 may exert a protective role in lipid metabolism in hepatocytes and adipocytes.

Targeting the HMGB1–CAV1 signaling axis represents a promising avenue for therapeutic intervention in metabolic disorders. Several studies have identified compounds that influence these pathways: (1) HMGB1 inhibitors: Glycyrrhizin, a natural HMGB1 antagonist, has been shown to attenuate oxidative stress, hepatic inflammation, and apoptosis in high fructose-fed rats, highlighting its therapeutic potential in liver dysfunction associated with metabolic syndrome [47]. Additionally, simvastatin has been reported to reduce vascular inflammation and atherosclerotic development in apolipoprotein E-deficient mice, potentially by interfering with HMGB1–RAGE signaling [48]. (2) CAV1 modulators: While direct pharmacological activators of CAV1 are limited, in vitro studies have demonstrated that cholesterol enrichment enhances the stabilization and membrane localization of CAV1 in COS-7 cells [49]. This suggests lipid composition may regulate CAV1 dynamics, which is relevant to adipocyte function and vascular health. Based on these findings, the therapeutic value of the HMGB1–CAV1 axis strategies in treating lipid disorders using HMGB1 inhibitors or CAV1 stabilizers is a topic worth investigating.

This study had certain limitations. The full scope of HMGB1 functions could not be verified in vivo, as HMGB1-knockout mice die from fatal neonatal hypoglycemia [26,50]. Additionally, these knockout mice exhibit significant forebrain underdevelopment during early embryonic stages [51]. This early postnatal lethality has substantially restricted research on HMGB1 in living organisms. Targeted HMGB1 silencing in primary adipocytes using siRNA offers a quick, cost-effective, and flexible approach that avoids breeding. However, it may not achieve complete suppression of HMGB1 like knockout mouse models and could cause off-target effects. The transient nature of siRNA silencing provides experimental flexibility but makes it harder to assess long-term metabolic effects, which may be more accurately studied with complete gene ablation. Future studies employing conditional HMGB1 deactivation in specific tissues or at defined developmental stages may help overcome these challenges. The results from the PEPPI computational program, a tool developed to predict protein–protein interactions [52], indicated no direct interaction between HMGB1 and CAV1. However, our immunoprecipitation assay revealed that the formation of the HMGB1–CAV1 complex was increased during adipocyte differentiation in a c-Src-dependent manner. These findings suggest that additional molecules may facilitate the formation of this complex. Therefore, further investigation using liquid chromatography-tandem mass spectrometry is warranted to identify the protein components within the co-immunoprecipitation complex.

## 4. Materials and Methods

### 4.1. Reagents

Recombinant human HMGB1 purified from *Escherichia coli* and mouse anti-α-tubulin antibodies were from Sigma-Aldrich (Saint Louis, MO, USA). Goat anti-HMGB1 and anti-aP2, mouse anti-PPARγ, anti-SREBP-1, anti-phospho-ERK, rabbit anti-HSL, anti-CAV1, and anti-ERK antibodies, as well as protein A/G beads, were from Santa Cruz Biotechnology (Santa Cruz, CA, USA). Rabbit anti-phospho-CAV1, anti-phospho-AMPK, anti-phospho-HSL (Ser563), anti-phospho-HSL (Ser660), and mouse anti-AMPK antibodies were obtained from Cell Signaling Technology (Danvers, MA, USA). PP1 (an inhibitor of Src-family tyrosine kinases) was from Tocris Bioscience (Bristol, UK). CL-316,243 (β3-adrenergic receptor agonist), the Glycerol Colorimetric Assay Kit, the Lipase Colorimetric Assay Kit, and the cAMP quantitative ELISA kit were from R&D Systems (Minneapolis, MN, USA).

### 4.2. Mice

The investigation conformed to the Guide for the Care and Use of Laboratory Animals published by the US National Institutes of Health (NIH Publication No. 85-23, revised 1996), and all animal experiments were approved by the Institutional Animal Care and Use Committee of National Chung Hsing University (No. 113-035). WT C57BL/6 mice were from the National Laboratory Animal Center of the National Institutes of Applied Research (Taipei, Taiwan). *CAV1*^−/−^ (B6.Cg-Cav1^tm1Mls^/J) mice were from Jackson Laboratory (Bar Harbor, ME, USA).

### 4.3. Cell Culture

Cells from the mouse preadipocyte cell line 3T3-L1 and isolated primary MEFs from WT or *CAV1*^−/−^ mice were cultured in Dulbecco’s modified Eagle’s medium (DMEM) containing 15% fetal bovine serum (FBS) and penicillin (100 U/mL)/streptomycin (100 μg/mL) in a humidified incubator at 37 °C, 95% air, and 5% CO_2_.

### 4.4. 3T3-L1 Preadipocyte Differentiation

Adipogenesis was initiated when 3T3-L1 cells reached full confluence. To induce differentiation, the growth medium was replaced with modified DMEM containing an adipogenic cocktail of 1-methyl-3-isobutylxanthine (0.5 mM), dexamethasone (0.5 μM), and insulin (1.7 μM). The fresh differentiation medium was provided daily throughout the induction phase. After three days, cells were transferred to standard DMEM without adipogenic factors and maintained for an additional 2 or 4 days to allow full maturation into adipocytes before subsequent experimental analyses.

### 4.5. MEF Isolation

Primary MEFs were isolated from WT and *CAV1*^−/−^ embryos at embryonic day 13.5. Following uterine extraction, embryos were dissected to remove brain tissue, internal organs, and embryonic membranes. The remaining tissue was mechanically minced and enzymatically digested with 0.25% trypsin-EDTA for 5 min at 37 °C with gentle shaking. The digestion was halted by adding DMEM supplemented with 10% FBS and antibiotics (100 U/mL penicillin, 100 μg/mL streptomycin). The cell suspension was centrifuged at 1000× *g* for 5 min at room temperature, and the pellet was resuspended in a growth medium before being plated in 100 mm culture dishes. Cultures were maintained with medium changes every 48 h until confluence. For adipogenic differentiation, confluent MEFs were treated with a differentiation medium containing adipogenic inducers for 4 days, then maintained in a standard growth medium without inducers for an additional 5 days. This protocol generated mature adipocytes suitable for subsequent experimental analyses.

### 4.6. RT-PCR

Total RNA was extracted using TRI Reagent (Sigma-Aldrich, Saint Louis, MO, USA). Cells were lysed at room temperature (25 °C) for 5 min, followed by vigorous mixing with chloroform and a 15-min incubation. After centrifugation (12,000× *g*, 15 min), the aqueous phase was collected and mixed with an equal volume of isopropanol. Following a 10-min incubation, RNA was pelleted by centrifugation (12,000× *g*, 10 min), washed with 75% ethanol, and centrifuged again (12,000× *g*, 5 min). The RNA pellet was air-dried, resuspended in DEPC-treated water, and heated at 60 °C for 15 min. For cDNA synthesis, 5 μg of RNA was reverse transcribed using the Superscript III system (Invitrogen, Waltham, MA, USA). RNA was combined with oligo(dT)12-18 primers (1 μL) and dNTPs (1 μL, 10 mM) in DEPC-treated water (10 μL total volume), heated to 65 °C for 5 min, and rapidly cooled on ice. The reaction was then supplemented with DTT (1 μL, 0.1 M), RNase Out (1 μL), 5X reaction buffer (4 μL), and Superscript III reverse transcriptase (1 μL). Reverse transcription proceeded at 50 °C for 60 min, followed by enzyme inactivation at 72 °C for 15 min. The cDNA products were then subjected to PCR reaction with the primers: HMGB1 (forward): 5′-GAG GAG CAT AAG AAG AAG CA-3′; HMGB1 (reverse): 5′-TTT GAT TTT GGG GCG GTA CT-3′; GAPDH (forward): 5′-TGT TCC AGT ATG ACT CCA CTC-3′; GAPDH (reverse): 5′-TCC ACC ACC CTG TTG CTG TA-3′. The thermal cycling conditions included an initial denaturation at 95 °C for 3 min, followed by 30 cycles of denaturation (95 °C, 30 s), annealing (57 °C, 30 s), and extension (72 °C, 60 s). A final extension step at 72 °C for 10 min was performed before storage at 4 °C. PCR products were analyzed on a 2% agarose gel, yielding expected fragment sizes of 269 bp (HMGB1) and 802 bp (GAPDH).

### 4.7. Preparation of Whole Cell Lysates

Cells were washed with phosphate-buffered saline (PBS) before lysis in a buffer containing 50 mM Tris (pH 7.5), 5 mM EDTA, and 300 mM NaCl. The lysis buffer was supplemented with 1% Triton X-100, protease inhibitors (10 μg/mL leupeptin and aprotinin, plus 1 mM phenylmethylsulfonyl fluoride), and phosphatase inhibitor cocktails 1 and 2 to preserve protein phosphorylation. The cell suspension was sonicated with a microprobe for 30 s on ice, followed by a 5-min incubation. Nuclear extracts were collected by centrifugation at 5000× *g* for 5 min at 4 °C, and the supernatant was retained for further analysis.

### 4.8. Western Blot Analysis

Cell lysates and nuclear extracts (50 µg per sample) underwent separation via sodium dodecyl sulfate (SDS)-PAGE using 8–12% gels, followed by transfer to Immobilon-P PVDF Membrane (Merck Millipore, Bedford, MA, USA). The membranes were blocked using a 5% non-fat milk solution at room temperature for an hour. Sequential incubation with primary antibodies and corresponding secondary antibodies was performed. The visualization of protein bands was achieved using an enhanced chemiluminescence kit (PerkinElmer, Waltham, MA, USA), with band intensity analyzed using ImageQuant 5.2 software (Healthcare Bio-Sciences, Philadelphia, PA, USA) for quantification.

### 4.9. Immunoprecipitation Assay

Cell lysate samples (1000 µg) were combined with specific primary antibodies and kept at 4 °C with gentle shaking overnight. The mixture was then supplemented with Protein A/G-Sepharose beads (20 µL) and further incubated for 2 h at 4 °C. The resulting complexes were pelleted by brief centrifugation (2000× *g*, 5 min) and underwent three washing cycles with PBS at 4 °C. Protein elution was performed using an SDS lysis buffer containing 1% Triton, 0.1% SDS, 0.2% sodium azide, and 0.5% sodium deoxycholate, supplemented with protease inhibitors (leupeptin and aprotinin, 10 µg/mL each). The eluted proteins were resolved using 8–12% SDS-PAGE gels, followed by membrane transfer and standard immunoblotting procedures such as western blot analysis.

### 4.10. Cell Proliferation

Cell proliferation was assessed using the MTT colorimetric assay. After overnight serum starvation in 24-well plates, cells were treated with rHMGB1 for 24 h. To evaluate viability, the MTT reagent was added to the culture medium at a final concentration of 0.5 mg/mL, and cells were incubated at 37 °C for 4 h. The medium was then removed, and formazan crystals were dissolved in 300 µL of isopropanol per well. Cell viability was quantified by measuring absorbance at 570 nm using a spectrophotometer.

### 4.11. Measurement of Glycerol Secretion and Cellular Triglyceride Levels

Glycerol levels in the media were measured using a commercial colorimetric detection kit. Cellular triglycerides were assessed using an enzymatic assay involving lipase-mediated colorimetric detection.

### 4.12. Measurement of Cellular cAMP Concentrations

Intracellular cAMP levels were quantified using ELISA. Cell lysates were diluted 1:2 and added to antibody-coated wells along with primary antibodies and cAMP conjugate. After a 3-h incubation at room temperature with gentle mixing, the plates were washed, and a substrate solution was added to initiate a chromogenic reaction, which was stopped after 30 min. Absorbance was measured at 450 and 570 nm, with final values calculated using a 2000 pmol/mL standard.

### 4.13. Statistical Analysis

Data are presented as the mean ± standard error of the mean (SEM). An unpaired *t*-test was used to compare two independent groups. The one-way ANOVA, followed by an LSD test, was performed to test multiple groups. SPSS software v20.0 (SPSS Inc., Chicago, IL, USA) was used for analysis. Differences were considered statistically significant at *p* < 0.05.

## 5. Conclusions

Our findings establish HMGB1 as a CAV1-regulated factor that inhibits lipolysis, unveiling a novel metabolic control mechanism in adipose tissue. This discovery fills critical knowledge gaps and positions HMGB1 signaling at the intersection of metabolism and inflammation in obesity. By elucidating the role of this alarmin in adipocyte function, we identify HMGB1 as a promising therapeutic target for tackling both metabolic disorders and cardiovascular disease. Interventions targeting the HMGB1 pathway may potentiate adipose tissue metabolic activity and improve metabolic health, offering a promising therapeutic avenue for millions affected by obesity-related conditions.

## Data Availability

Data are contained within the article.
